# *N*-Acetyl-4-(benzene­sulfonamido)benzene­sulfonamide

**DOI:** 10.1107/S1600536809015876

**Published:** 2009-04-30

**Authors:** Muhammad Ashfaq, M. Nawaz Tahir, Islam Ullah Khan, Muhammad Nadeem Arshad, Syed Saeed-ul-Hassan

**Affiliations:** a Department of Chemistry, Government College University, Lahore, Pakistan; b Department of Physics, University of Sargodha, Sagrodha, Pakistan; c University College of Pharmacy, University of the Punjab, Lahore, Pakistan

## Abstract

In the mol­ecule of the title compound, C_14_H_14_N_2_O_5_S_2_, the dihedral angle between the aromatic rings is 77.75 (9)°. The acetamide group is planar [maximum deviation = 0.002 (3) Å] and oriented at dihedral angles of 13.49 (21) and 73.94 (10)° with respect to the aromatic rings. An intra­molecular C—H⋯O inter­action results in the formation of a six-membered ring. In the crystal structure, inter­molecular N—H⋯O and C—H⋯O inter­actions link the mol­ecules into a three-dimensional network, forming *R*_2_^2^(20) ring motifs.

## Related literature

For related structures, see: Chohan *et al.* (2008 ▶, 2009 ▶); Deng & Mani (2006 ▶); Ellingboe *et al.* (1992 ▶); Shad *et al.* (2009 ▶); Tahir *et al.* (2008 ▶). For ring-motifs, see: Bernstein *et al.* (1995 ▶).
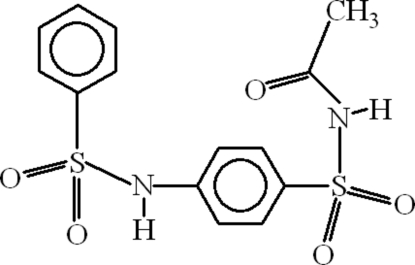


## Experimental

### 

#### Crystal data

C_14_H_14_N_2_O_5_S_2_
*M* *_r_* = 354.39
Monoclinic, 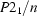

*a* = 9.9316 (9) Å
*b* = 9.4828 (8) Å
*c* = 17.6490 (17) Å
β = 103.169 (5)°
*V* = 1618.5 (3) Å^3^
*Z* = 4
Mo *K*α radiation
μ = 0.36 mm^−1^
*T* = 296 K
0.28 × 0.22 × 0.18 mm

#### Data collection

Bruker Kappa APEXII CCD area-detector diffractometer
Absorption correction: multi-scan (*SADABS*; Bruker, 2005 ▶) *T*_min_ = 0.909, *T*_max_ = 0.940
17674 measured reflections
4034 independent reflections
2423 reflections with *I* > 2σ(*I*)
*R*_int_ = 0.060

#### Refinement

*R*[*F* ^2^ > 2σ(*F* ^2^)] = 0.049
*wR*(*F* ^2^) = 0.128
*S* = 1.02
4034 reflections
209 parameters
H-atom parameters constrained
Δρ_max_ = 0.31 e Å^−3^
Δρ_min_ = −0.36 e Å^−3^

### 

Data collection: *APEX2* (Bruker, 2007 ▶); cell refinement: *SAINT* (Bruker, 2007 ▶); data reduction: *SAINT*; program(s) used to solve structure: *SHELXS97* (Sheldrick, 2008 ▶); program(s) used to refine structure: *SHELXL97* (Sheldrick, 2008 ▶); molecular graphics: *ORTEP-3 for Windows* (Farrugia, 1997 ▶) and *PLATON* (Spek, 2009 ▶); software used to prepare material for publication: *WinGX* (Farrugia, 1999 ▶) and *PLATON*.

## Supplementary Material

Crystal structure: contains datablocks global, I. DOI: 10.1107/S1600536809015876/hk2676sup1.cif

Structure factors: contains datablocks I. DOI: 10.1107/S1600536809015876/hk2676Isup2.hkl

Additional supplementary materials:  crystallographic information; 3D view; checkCIF report

## Figures and Tables

**Table 1:** Hydrogen-bond geometry (Å, °)

*D*—H⋯*A*	*D*—H	H⋯*A*	*D*⋯*A*	*D*—H⋯*A*
N1—H1*N*⋯O5^i^	0.86	2.25	2.839 (3)	126
N2—H2*N*⋯O1^ii^	0.86	2.14	2.922 (3)	151
C8—H8⋯O2	0.93	2.49	3.116 (3)	125
C9—H9⋯O4^iii^	0.93	2.60	3.237 (3)	126
C14—H14*A*⋯O2^iv^	0.96	2.56	3.401 (4)	147

Symmetry codes: (i) 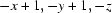; (ii) 
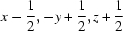
; (iii) 
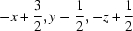
; (iv) 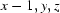.

**Fig. 1.**
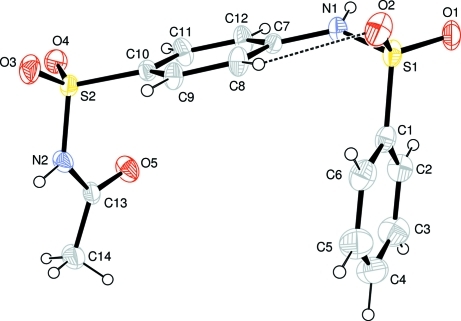
The molecular structure of the title molecule, with the atom-numbering scheme. Displacement ellipsoids are drawn at the 50% probability level. Hydrogen bond is shown as dashed line.

**Fig. 2.**
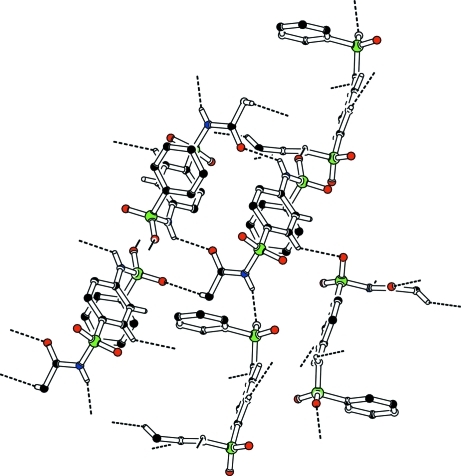
A partial packing diagram of the title compound. Hydrogen bonds are shown as dashed lines.

## supplementary crystallographic information

### Comment

Sulfonamides have attracted much attention, due to their extensive use in medicine. We have reported the syntheses and crystal structures of sulfonamides, which have the central portion of title compound as common (Chohan *et al.*, 2008, 2009; Shad *et al.*, 2009; Tahir *et al.*, 2008). Similarly, the crystal structure of *N*-Methyl-*N*-(2-(methyl(1-methyl-1*H*-benzimidazol-2-yl)amino)- ethyl)-4-((methylsulfonyl)amino)-benzenesulfonamide (Ellingboe *et al.*, 1992) has been reported, which also has a central portion as in the title compound.

In the molecule of the title compound (Fig 1), rings A (C1-C6) and B (C7-C12) are, of course, planar. The acetamide moiety C (N2/O5/C13/C14) is also planar with a maximum deviation of 0.002 (3) Å for atom C13. The diheadral angles between them are A/B = 77.75 (9), A/C = 13.49 (21) and B/C = 73.94 (10) °. The SO_2_ groups are oriented at a dihedral angle of 71.02 (15)°. Intramolecular C-H···O interaction (Table 1) results in the formation of a six-membered ring D (S1/O2/N1/C7/C8/H8) having twisted conformation.

In the crystal structure, intermolecular N-H···O and C-H···O interactions (Table 1) link the molecules into a three-dimensional network forming *R*_2_^2^(20) ring motifs (Bernstein *et al.*, 1995), in which they may be effective in the stabilization of the structure.

### Experimental

The title compound was synthesized according to a literature method (Deng & Mani, 2006). For the preparation of the title compound, phenylglycine (2 g, 5.3 mmol) was dissolved in distilled water, and then benzene sulfonyl chloride (0.93 g, 5.3 mmol) was added. It was stirred at room temperature. During the reaction pH was maintained at 8-9, strictly using Na_2_CO_3_ (1 M), since HCl was produced as a byproduct, which lowers the pH. The completion of reaction was observed by the consumption of the oily drops of benzene sulfonyl chloride. On completion, pH was adjusted to 2-3 using HCl (2 N). The precipitate formed was filtered, washed with distilled water and recrystalyzed from methanol.

### Refinement

H atoms were positioned geometrically, with N-H = 0.86 Å (for NH) and C-H = 0.93 and 0.96 Å for aromatic and methyl H, respectively, and constrained to ride on their parent atoms, with U_iso_(H) = xU_eq_(C,N), where x = 1.5 for methyl H and x = 1.2 for all other H atoms.

### Crystal data

**Table:**

C_14_H_14_N_2_O_5_S_2_	*F*(000) = 736
*M**_r_* = 354.39	*D*_x_ = 1.454 Mg m^−^^3^
Monoclinic, *P*2_1_/*n*	Mo *K*α radiation, λ = 0.71073 Å
Hall symbol: -P 2yn	Cell parameters from 4034 reflections
*a* = 9.9316 (9) Å	θ = 2.4–28.3°
*b* = 9.4828 (8) Å	µ = 0.36 mm^−^^1^
*c* = 17.6490 (17) Å	*T* = 296 K
β = 103.169 (5)°	Prism, colorless
*V* = 1618.5 (3) Å^3^	0.28 × 0.22 × 0.18 mm
*Z* = 4	

### Data collection

**Table:**

Bruker Kappa APEXII CCD area-detector diffractometer	4034 independent reflections
Radiation source: fine-focus sealed tube	2423 reflections with *I* > 2σ(*I*)
graphite	*R*_int_ = 0.060
Detector resolution: 7.40 pixels mm^-1^	θ_max_ = 28.3°, θ_min_ = 2.4°
ω scans	*h* = −13→13
Absorption correction: multi-scan (*SADABS*; Bruker, 2005)	*k* = −11→12
*T*_min_ = 0.909, *T*_max_ = 0.940	*l* = −22→23
17674 measured reflections	

### Refinement

**Table:**

Refinement on *F*^2^	Primary atom site location: structure-invariant direct methods
Least-squares matrix: full	Secondary atom site location: difference Fourier map
*R*[*F*^2^ > 2σ(*F*^2^)] = 0.049	Hydrogen site location: inferred from neighbouring sites
*wR*(*F*^2^) = 0.128	H-atom parameters constrained
*S* = 1.02	*w* = 1/[σ^2^(*F*_o_^2^) + (0.052*P*)^2^ + 0.3831*P*] where *P* = (*F*_o_^2^ + 2*F*_c_^2^)/3
4034 reflections	(Δ/σ)_max_ < 0.001
209 parameters	Δρ_max_ = 0.31 e Å^−^^3^
0 restraints	Δρ_min_ = −0.36 e Å^−^^3^

### Special details

**Table:**

Geometry. Bond distances, angles *etc*. have been calculated using the rounded fractional coordinates. All su's are estimated from the variances of the (full) variance-covariance matrix. The cell e.s.d.'s are taken into account in the estimation of distances, angles and torsion angles
Refinement. Refinement of *F*^2^ against ALL reflections. The weighted *R*-factor *wR* and goodness of fit *S* are based on *F*^2^, conventional *R*-factors *R* are based on *F*, with *F* set to zero for negative *F*^2^. The threshold expression of *F*^2^ > σ(*F*^2^) is used only for calculating *R*-factors(gt) *etc*. and is not relevant to the choice of reflections for refinement. *R*-factors based on *F*^2^ are statistically about twice as large as those based on *F*, and *R*- factors based on ALL data will be even larger.

### Fractional atomic coordinates and isotropic or equivalent isotropic displacement parameters (Å^2^)

**Table:**

	*x*	*y*	*z*	*U*_iso_*/*U*_eq_	
S1	0.90183 (7)	0.28463 (8)	−0.04571 (4)	0.0407 (3)	
S2	0.58491 (7)	0.45284 (7)	0.26062 (4)	0.0374 (2)	
O1	0.9231 (2)	0.3308 (2)	−0.11946 (11)	0.0530 (7)	
O2	1.01569 (19)	0.2335 (2)	0.01166 (11)	0.0546 (7)	
O3	0.68083 (19)	0.4029 (2)	0.32790 (10)	0.0564 (7)	
O4	0.5257 (2)	0.58953 (19)	0.26098 (11)	0.0510 (7)	
O5	0.3371 (2)	0.4255 (2)	0.13539 (11)	0.0560 (7)	
N1	0.8361 (2)	0.4215 (2)	−0.01260 (12)	0.0384 (7)	
N2	0.4595 (2)	0.3347 (2)	0.24701 (12)	0.0387 (7)	
C1	0.7705 (3)	0.1563 (3)	−0.06104 (15)	0.0396 (9)	
C2	0.6519 (3)	0.1794 (3)	−0.11844 (17)	0.0518 (11)	
C3	0.5482 (3)	0.0815 (4)	−0.1295 (2)	0.0703 (14)	
C4	0.5621 (4)	−0.0385 (4)	−0.0833 (3)	0.0759 (17)	
C5	0.6797 (4)	−0.0586 (3)	−0.0265 (2)	0.0729 (16)	
C6	0.7849 (3)	0.0381 (3)	−0.01472 (18)	0.0545 (11)	
C7	0.7799 (2)	0.4241 (3)	0.05379 (14)	0.0315 (8)	
C8	0.8129 (3)	0.3261 (3)	0.11352 (15)	0.0417 (9)	
C9	0.7502 (3)	0.3347 (3)	0.17563 (14)	0.0389 (9)	
C10	0.6584 (3)	0.4411 (3)	0.17997 (14)	0.0321 (8)	
C11	0.6272 (3)	0.5403 (3)	0.12140 (17)	0.0472 (10)	
C12	0.6873 (3)	0.5305 (3)	0.05858 (16)	0.0446 (10)	
C13	0.3439 (3)	0.3407 (3)	0.18721 (15)	0.0393 (9)	
C14	0.2314 (3)	0.2385 (4)	0.19149 (18)	0.0655 (13)	
H1N	0.83546	0.49926	−0.03774	0.0461*	
H2	0.64289	0.26019	−0.14902	0.0622*	
H2N	0.46714	0.26548	0.27920	0.0464*	
H3	0.46813	0.09538	−0.16798	0.0843*	
H4	0.49166	−0.10523	−0.09098	0.0910*	
H5	0.68825	−0.13877	0.00460	0.0874*	
H6	0.86471	0.02409	0.02393	0.0655*	
H8	0.87684	0.25522	0.11176	0.0501*	
H9	0.77038	0.26763	0.21503	0.0467*	
H11	0.56602	0.61331	0.12432	0.0566*	
H12	0.66522	0.59658	0.01874	0.0536*	
H14A	0.19294	0.20141	0.14056	0.0984*	
H14B	0.16038	0.28576	0.21067	0.0984*	
H14C	0.26868	0.16277	0.22601	0.0984*	

### Atomic displacement parameters (Å^2^)

**Table:**

	*U*^11^	*U*^22^	*U*^33^	*U*^12^	*U*^13^	*U*^23^
S1	0.0359 (4)	0.0556 (5)	0.0317 (4)	0.0099 (3)	0.0102 (3)	−0.0074 (3)
S2	0.0349 (4)	0.0507 (4)	0.0281 (3)	−0.0047 (3)	0.0104 (3)	−0.0067 (3)
O1	0.0571 (13)	0.0707 (14)	0.0371 (11)	0.0023 (11)	0.0232 (10)	−0.0102 (10)
O2	0.0387 (11)	0.0718 (14)	0.0482 (12)	0.0218 (10)	−0.0006 (10)	−0.0091 (10)
O3	0.0407 (11)	0.0984 (16)	0.0271 (10)	−0.0047 (11)	0.0013 (9)	−0.0017 (10)
O4	0.0557 (12)	0.0467 (11)	0.0586 (13)	−0.0019 (10)	0.0294 (10)	−0.0167 (10)
O5	0.0474 (12)	0.0781 (15)	0.0391 (11)	−0.0021 (11)	0.0025 (10)	0.0205 (11)
N1	0.0451 (13)	0.0396 (12)	0.0341 (12)	0.0070 (10)	0.0163 (10)	0.0013 (10)
N2	0.0379 (12)	0.0473 (13)	0.0291 (12)	−0.0050 (10)	0.0042 (10)	0.0113 (10)
C1	0.0409 (15)	0.0424 (15)	0.0356 (15)	0.0128 (13)	0.0089 (12)	−0.0048 (12)
C2	0.0467 (18)	0.0586 (19)	0.0480 (18)	0.0087 (15)	0.0062 (15)	0.0005 (15)
C3	0.046 (2)	0.085 (3)	0.074 (2)	0.000 (2)	0.0015 (18)	−0.008 (2)
C4	0.072 (3)	0.066 (3)	0.095 (3)	−0.016 (2)	0.030 (2)	−0.021 (2)
C5	0.093 (3)	0.046 (2)	0.082 (3)	0.002 (2)	0.025 (2)	0.0040 (18)
C6	0.063 (2)	0.0479 (18)	0.0515 (19)	0.0132 (16)	0.0109 (16)	−0.0028 (15)
C7	0.0290 (13)	0.0378 (14)	0.0279 (13)	−0.0014 (11)	0.0067 (10)	−0.0040 (11)
C8	0.0456 (16)	0.0467 (16)	0.0347 (15)	0.0180 (13)	0.0130 (13)	0.0028 (12)
C9	0.0460 (16)	0.0421 (15)	0.0291 (14)	0.0109 (13)	0.0095 (12)	0.0051 (12)
C10	0.0313 (13)	0.0358 (14)	0.0306 (13)	0.0013 (11)	0.0098 (11)	−0.0013 (11)
C11	0.0574 (18)	0.0407 (16)	0.0517 (18)	0.0180 (14)	0.0298 (15)	0.0084 (13)
C12	0.0578 (18)	0.0403 (16)	0.0420 (16)	0.0159 (14)	0.0242 (14)	0.0124 (12)
C13	0.0376 (15)	0.0549 (17)	0.0272 (14)	−0.0049 (13)	0.0109 (12)	0.0006 (12)
C14	0.0526 (19)	0.100 (3)	0.0427 (18)	−0.0311 (19)	0.0083 (15)	0.0000 (17)

### Geometric parameters (Å, °)

**Table:**

S1—O1	1.434 (2)	C7—C12	1.381 (4)
S1—O2	1.420 (2)	C7—C8	1.387 (4)
S1—N1	1.621 (2)	C8—C9	1.381 (4)
S1—C1	1.759 (3)	C9—C10	1.374 (4)
S2—O3	1.4235 (19)	C10—C11	1.380 (4)
S2—O4	1.424 (2)	C11—C12	1.377 (4)
S2—N2	1.652 (2)	C13—C14	1.494 (5)
S2—C10	1.745 (3)	C2—H2	0.9300
O5—C13	1.208 (3)	C3—H3	0.9300
N1—C7	1.408 (3)	C4—H4	0.9300
N2—C13	1.372 (3)	C5—H5	0.9300
N1—H1N	0.8600	C6—H6	0.9300
N2—H2N	0.8600	C8—H8	0.9300
C1—C6	1.375 (4)	C9—H9	0.9300
C1—C2	1.385 (4)	C11—H11	0.9300
C2—C3	1.367 (5)	C12—H12	0.9300
C3—C4	1.388 (6)	C14—H14A	0.9600
C4—C5	1.368 (6)	C14—H14B	0.9600
C5—C6	1.370 (5)	C14—H14C	0.9600
			
S1···H8	2.8600	C8···C6	3.517 (4)
O1···N2^i^	2.922 (3)	C9···O4^xi^	3.237 (3)
O2···C6^ii^	3.242 (4)	C10···O5	3.111 (4)
O2···C5^ii^	3.407 (4)	C11···O5	3.142 (4)
O2···C8	3.116 (3)	C12···O5^vi^	3.402 (3)
O2···C14^iii^	3.401 (4)	C14···O4^xii^	3.193 (4)
O4···O5	2.992 (3)	C14···O2^xiii^	3.401 (4)
O4···C8^iv^	3.300 (3)	C2···H14B^i^	3.0500
O4···C9^iv^	3.237 (3)	C2···H11^vi^	2.9100
O4···C14^v^	3.193 (4)	C5···H14A^x^	2.9400
O5···C11	3.142 (4)	C6···H8	3.0200
O5···N1^vi^	2.839 (3)	H1N···H12	2.3400
O5···O4	2.992 (3)	H1N···O2^vii^	2.9200
O5···C12^vi^	3.402 (3)	H1N···O5^vi^	2.2500
O5···C10	3.111 (4)	H2···O1	2.7900
O1···H14C^i^	2.8100	H2···O4^vi^	2.6900
O1···H2	2.7900	H2···H11^vi^	2.5200
O1···H2N^i^	2.1400	H2···H14B^i^	2.5600
O2···H8	2.4900	H2N···H14C	2.2100
O2···H6^ii^	2.8500	H2N···O1^ix^	2.1400
O2···H6	2.5300	H3···O3^xiv^	2.8400
O2···H1N^vii^	2.9200	H5···H12^xv^	2.5400
O2···H14A^iii^	2.5600	H6···O2	2.5300
O3···H3^viii^	2.8400	H6···O2^ii^	2.8500
O3···H9	2.6900	H8···S1	2.8600
O4···H11	2.5400	H8···O2	2.4900
O4···H14B^v^	2.7500	H8···C6	3.0200
O4···H2^vi^	2.6900	H8···O4^xi^	2.7300
O4···H8^iv^	2.7300	H9···O3	2.6900
O4···H9^iv^	2.6000	H9···O4^xi^	2.6000
O5···H1N^vi^	2.2500	H11···O4	2.5400
O5···H12^vi^	2.7200	H11···C2^vi^	2.9100
N1···O5^vi^	2.839 (3)	H11···H2^vi^	2.5200
N2···O1^ix^	2.922 (3)	H12···H1N	2.3400
C1···C8	3.416 (4)	H12···H5^xvi^	2.5400
C4···C5^x^	3.534 (6)	H12···O5^vi^	2.7200
C4···C4^x^	3.515 (7)	H14A···O2^xiii^	2.5600
C5···O2^ii^	3.407 (4)	H14A···C5^x^	2.9400
C5···C4^x^	3.534 (6)	H14B···O4^xii^	2.7500
C6···C8	3.517 (4)	H14B···C2^ix^	3.0500
C6···O2^ii^	3.242 (4)	H14B···H2^ix^	2.5600
C8···O2	3.116 (3)	H14C···H2N	2.2100
C8···C1	3.416 (4)	H14C···O1^ix^	2.8100
C8···O4^xi^	3.300 (3)		
			
O1—S1—O2	119.56 (12)	S2—C10—C11	120.2 (2)
O1—S1—N1	103.74 (11)	C9—C10—C11	119.9 (3)
O1—S1—C1	109.21 (12)	C10—C11—C12	119.5 (3)
O2—S1—N1	109.70 (11)	C7—C12—C11	121.0 (3)
O2—S1—C1	108.39 (13)	O5—C13—C14	123.8 (3)
N1—S1—C1	105.31 (12)	N2—C13—C14	116.0 (2)
O3—S2—O4	119.85 (12)	O5—C13—N2	120.2 (3)
O3—S2—N2	103.58 (11)	C1—C2—H2	121.00
O3—S2—C10	109.54 (13)	C3—C2—H2	120.00
O4—S2—N2	108.64 (11)	C2—C3—H3	120.00
O4—S2—C10	108.16 (13)	C4—C3—H3	120.00
N2—S2—C10	106.25 (12)	C3—C4—H4	120.00
S1—N1—C7	125.64 (18)	C5—C4—H4	120.00
S2—N2—C13	123.70 (18)	C4—C5—H5	120.00
C7—N1—H1N	117.00	C6—C5—H5	120.00
S1—N1—H1N	117.00	C1—C6—H6	121.00
S2—N2—H2N	118.00	C5—C6—H6	121.00
C13—N2—H2N	118.00	C7—C8—H8	120.00
S1—C1—C2	118.7 (2)	C9—C8—H8	120.00
S1—C1—C6	120.0 (2)	C8—C9—H9	120.00
C2—C1—C6	121.3 (3)	C10—C9—H9	120.00
C1—C2—C3	119.0 (3)	C10—C11—H11	120.00
C2—C3—C4	120.1 (3)	C12—C11—H11	120.00
C3—C4—C5	119.9 (3)	C7—C12—H12	119.00
C4—C5—C6	120.8 (3)	C11—C12—H12	120.00
C1—C6—C5	118.9 (3)	C13—C14—H14A	109.00
C8—C7—C12	119.2 (2)	C13—C14—H14B	109.00
N1—C7—C8	123.4 (2)	C13—C14—H14C	109.00
N1—C7—C12	117.4 (2)	H14A—C14—H14B	109.00
C7—C8—C9	119.5 (3)	H14A—C14—H14C	110.00
C8—C9—C10	120.8 (2)	H14B—C14—H14C	109.00
S2—C10—C9	119.9 (2)		
			
O1—S1—N1—C7	171.1 (2)	S2—N2—C13—O5	9.3 (4)
O2—S1—N1—C7	−60.1 (2)	S2—N2—C13—C14	−170.4 (2)
C1—S1—N1—C7	56.4 (2)	S1—C1—C2—C3	−178.6 (2)
O1—S1—C1—C2	−44.2 (3)	C6—C1—C2—C3	−0.8 (4)
O1—S1—C1—C6	137.9 (2)	S1—C1—C6—C5	178.4 (2)
O2—S1—C1—C2	−176.0 (2)	C2—C1—C6—C5	0.5 (5)
O2—S1—C1—C6	6.1 (3)	C1—C2—C3—C4	0.4 (5)
N1—S1—C1—C2	66.7 (3)	C2—C3—C4—C5	0.1 (6)
N1—S1—C1—C6	−111.2 (2)	C3—C4—C5—C6	−0.4 (6)
O3—S2—N2—C13	−179.6 (2)	C4—C5—C6—C1	0.0 (5)
O4—S2—N2—C13	52.0 (2)	N1—C7—C8—C9	−178.4 (2)
C10—S2—N2—C13	−64.2 (2)	C12—C7—C8—C9	1.5 (4)
O3—S2—C10—C9	32.2 (3)	N1—C7—C12—C11	179.7 (3)
O3—S2—C10—C11	−146.2 (2)	C8—C7—C12—C11	−0.2 (4)
O4—S2—C10—C9	164.5 (2)	C7—C8—C9—C10	−1.7 (4)
O4—S2—C10—C11	−14.0 (3)	C8—C9—C10—S2	−177.9 (2)
N2—S2—C10—C9	−79.1 (3)	C8—C9—C10—C11	0.5 (4)
N2—S2—C10—C11	102.5 (2)	S2—C10—C11—C12	179.2 (2)
S1—N1—C7—C8	22.2 (3)	C9—C10—C11—C12	0.8 (4)
S1—N1—C7—C12	−157.8 (2)	C10—C11—C12—C7	−1.0 (4)

Symmetry codes: (i) *x*+1/2, −*y*+1/2, *z*−1/2; (ii) −*x*+2, −*y*, −*z*; (iii) *x*+1, *y*, *z*; (iv) −*x*+3/2, *y*+1/2, −*z*+1/2; (v) −*x*+1/2, *y*+1/2, −*z*+1/2; (vi) −*x*+1, −*y*+1, −*z*; (vii) −*x*+2, −*y*+1, −*z*; (viii) *x*+1/2, −*y*+1/2, *z*+1/2; (ix) *x*−1/2, −*y*+1/2, *z*+1/2; (x) −*x*+1, −*y*, −*z*; (xi) −*x*+3/2, *y*−1/2, −*z*+1/2; (xii) −*x*+1/2, *y*−1/2, −*z*+1/2; (xiii) *x*−1, *y*, *z*; (xiv) *x*−1/2, −*y*+1/2, *z*−1/2; (xv) *x*, *y*−1, *z*; (xvi) *x*, *y*+1, *z*.

### Hydrogen-bond geometry (Å, °)

**Table:**

*D*—H···*A*	*D*—H	H···*A*	*D*···*A*	*D*—H···*A*
N1—H1N···O5^vi^	0.86	2.25	2.839 (3)	126
N2—H2N···O1^ix^	0.86	2.14	2.922 (3)	151
C8—H8···O2	0.93	2.49	3.116 (3)	125
C9—H9···O4^xi^	0.93	2.60	3.237 (3)	126
C14—H14A···O2^xiii^	0.96	2.56	3.401 (4)	147

Symmetry codes: (vi) −*x*+1, −*y*+1, −*z*; (ix) *x*−1/2, −*y*+1/2, *z*+1/2; (xi) −*x*+3/2, *y*−1/2, −*z*+1/2; (xiii) *x*−1, *y*, *z*.
